# MalVac: Database of malarial vaccine candidates

**DOI:** 10.1186/1475-2875-7-184

**Published:** 2008-09-23

**Authors:** Rupanjali Chaudhuri, Shakil Ahmed, Faraz Alam Ansari, Harinder Vir Singh, Srinivasan Ramachandran

**Affiliations:** 1 G.N Ramachandran Knowledge Centre for Genome Informatics, Institute of Genomics and Integrative Biology (Council of Scientific and Industrial Research), Mall Road, Delhi 110007, India

## Abstract

**Background:**

The sequencing of genomes of the Plasmodium species causing malaria, offers immense opportunities to aid in the development of new therapeutics and vaccine candidates through Bioinformatics tools and resources.

**Methods:**

The starting point of MalVac database is the collection of known vaccine candidates and a set of predicted vaccine candidates identified from the whole proteome sequences of Plasmodium species provided by PlasmoDb 5.4 release (31st October 2007). These predicted vaccine candidates are the adhesins and adhesin-like proteins from Plasmodium species, *Plasmodium falciparum*, *Plasmodium vivax *and *Plasmodium yoelii*. Subsequently, these protein sequences were analysed through 20 publicly available algorithms to obtain Orthologs, Paralogs, BetaWraps, TargetP, TMHMM, SignalP, CDDSearch, BLAST with Human Ref. Proteins, T-cell epitopes, B-cell epitopes, Discotopes, and allergen predictions. All of this information was collected and organized with the ORFids of the protein sequences as primary keys. This information is relevant from the view point of Reverse Vaccinology in facilitating decision making on the most probable choice for vaccine strategy.

**Results:**

Detailed information on the patterning of the epitopes and other motifs of importance from the viewpoint of reverse vaccinology has been obtained on the most probable protein candidates for vaccine investigation from three major malarial species. Analysis data are available on 161 adhesin proteins from *P. falciparum*, 137 adhesin proteins from *P. vivax *and 34 adhesin proteins from *P. yoelii*. The results are displayed in convenient tabular format and a facility to export the entire data has been provided. The MalVac database is a "community resource". Users are encouraged to export data and further contribute by value addition. Value added data may be sent back to the community either through MalVac or PlasmoDB.

**Conclusion:**

A web server MalVac for facilitation of the identification of probable vaccine candidates has been developed and can be freely accessed.

## Background

Malaria is a major killer disease. Annually, about 500 million people get infected and an estimated 1 million deaths occur. Despite numerous efforts we still do not have effective vaccines [1]. Among the parasites that cause malaria, the most common and widely distributed is *Plasmodium vivax *[2]. But the most fatal form of malaria is caused by *Plasmodium falciparum *[3]. *Plasmodium yoelii *is a commonly used rodent malaria parasite as a model to study malaria infection. Malaria caused by *P. yoelii *has similarities to that caused by *P. falciparum *and *P. vivax *[4].

Currently, several vaccines against multiple stages are in clinical development, including pre-erythrocytic, blood stage and others [3]. Although these advancements raise the hopes of the availability of an effective vaccine, it is noted that our limited knowledge on the details of the immune responses is becoming a major handicap [1]. The availability of complete genome sequences of *Plasmodium falciparum *[5], *P. yoelii *[4] and *P. vivax *has provided new opportunity for applying the principles of Reverse Vaccinology. Reverse vaccinology uses bioinformatics in the initial steps to identify potential antigens, which are subsequently examined for their efficacy and toxicity. In its maiden application, use of algorithm for prediction of sub-cellular location boosted the power of identifying potential vaccine candidates [6]. Subsequently, enhancements have been proposed to reverse vaccinology by suggesting the use of additional algorithms to find probability of being an adhesin, of topology (transmembrane regions) and to find similarity with host protein [7].

Recently, integrative approaches are proposed for Reverse Vaccinology by including prediction of multiple features of proteins [8]. Adopting this strategy, the following predictions were incorporated: of adhesins [9] and their orthologs [10], paralogs [11], transmembrane topologies [12], beta helix supersecondary structural motifs [13], subcellular localization [14,15], similarity against Human proteins [16], antigenic regions [17], conserved domains[18], epitopes [19-26] and allergens [27-29]. The work flow started with adhesin prediction algorithm, which holds an important position in vaccine development process. The adhesin proteins mediate the adherence of malaria parasites to the host cells and facilitate invasion. Targeting these adhesins to abrogate the colonization process can prevent malaria infection [9,30].

The multiple features of potential vaccine candidates coupled with information on the current candidates being pursued can be queried through a user friendly interface. These data are housed in MalVac database, which can aid in the discovery of adhesin based vaccines.

## Methods

### Database architecture

**Figure 1:**
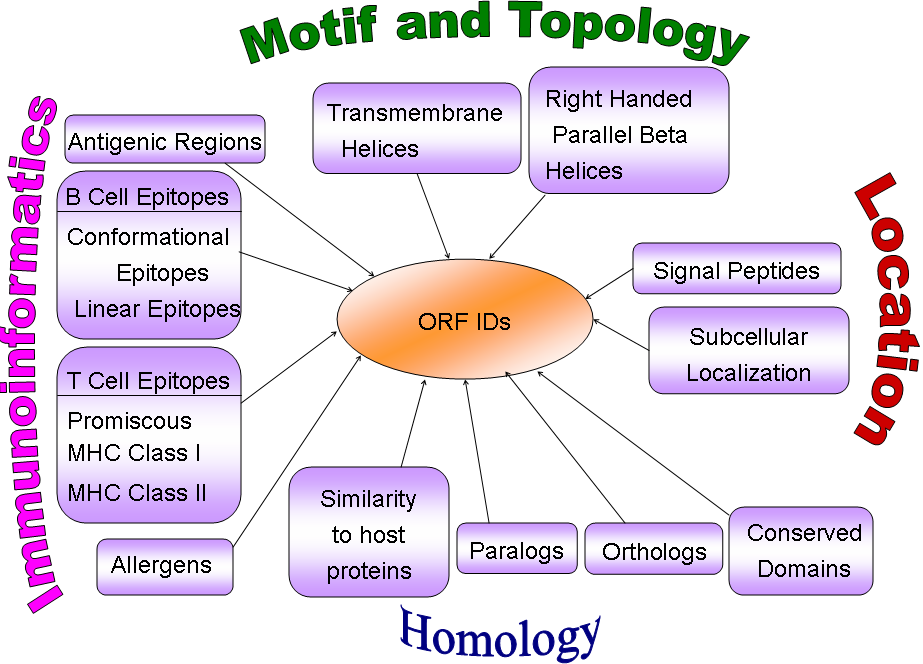
**The MalVac layout.** All data are organized in relation to the primary key ORF ID.

The ORF identification tags (ORF ID) assigned to proteins of malaria parasites as given in PlasmoDB 5.4 release of 31^st ^October 2007 [31] were used as primary keys. The database was developed using MySQL version 4.1.20 at back end and operated in Red Hat Enterprise Linux ES release 4. The web interfaces have been developed in HTML and PHP 5.1.4, which dynamically execute the MySQL queries to fetch the stored data and is run through Apache2 server. The overall layout of MalVac is provided in Figure 1.

The first step towards MalVac database creation is the collection of known vaccine candidates and a set of predicted vaccine candidates identified from the whole proteome sequences of Plasmodium species provided by PlasmoDB 5.4 release(31st October 2007). These predicted vaccine candidates are the adhesins and adhesin-like proteins from Plasmodium species, *P. falciparum*, *P. vivax *and *P. yoelii *using MAAP server [9]. Subsequently these protein sequences were analysed with 20 algorithms important from the view of reverse vaccinology (Table 1).

**Table 1:** Algorithms used to predict molecular features of potential malarial vaccine candidates and housed in MalVac.

Algorithm	Principle	Role in MalVac	Reference
1. MAAP	Predicts Malarial adhesins and adhesins-like proteins based on Support Vector Machines	Adhesin and Adhesin like protein prediction.	[9]
2. BLASTCLUST	Clusters protein or DNA sequences based on pair wise matches found using the BLAST algorithm in case of proteins or Mega BLAST algorithm for DNA.	Paralogs finding	[11]
3. TMHMM Server v. 2.0	Predicts the transmembrane helices in proteins based on Hidden Markov Model.	Transmembrane helices prediction	[12]
4. BetaWrap	Predicts the right-handed parallel beta-helix supersecondary structural motif in primary amino acid sequences by using beta-strand interactions learned from non-beta-helix structures.	Betawrap finding	[13]
5. TargetP1.1	Predicts the subcellular location of eukaryotic proteins based on the predicted presence of any of the N-terminal presequences: chloroplast transit peptide (**cTP**), mitochondrial targeting peptide (**mTP**) or secretory pathway signal peptide (**SP**).	Localization Prediction.	[14]
6. SignalP 3.0	Predicts the presence and location of signal peptide cleavage sites in amino acid sequences from different organisms. The method incorporates a prediction of cleavage sites and a signal peptide/non-signal peptide prediction based on a combination of several artificial neural networks and hidden Markov models.	Signal Peptide Prediction.	[15]
7. BlastP	It uses the BLAST algorithm to compare an amino acid query sequence against a protein sequence database.	Prediction of similarity to human reference proteins.	[16]
8. Antigenic	Predicts potentially antigenic regions of a protein sequence, based on occurrence frequencies of amino acid residue types in known epitopes.	Antigenic region prediction.	[17]
9. Conserved Domain Database and Search Service, v2.13	The Database is a collection of multiple sequence alignments for ancient domains and full-length proteins. It is used to identify the conserved domains present in a protein query sequence.	Conserved Domain Finding	[18]
10. ABCPred	Predict *B cell epitope(s) *in an antigen sequence, using artificial neural network.	Linear B Cell Epitope Prediction.	[19]
11. BcePred	Predicts linear B-cell epitopes, using physico-chemical properties.	Linear B Cell Epitope Prediction.	[20]
12. Discotope 1.1	Predicts discontinuous B cell epitopes from protein three dimensional structures utilizing calculation of surface accessibility (estimated in terms of contact numbers) and a novel epitope propensity amino acid score.	Conformational B Cell Epitope Prediction.	[21]
13. CEP	The algorithm predicts epitopes of protein antigens with known structures. It uses accessibility of residues and spatial distance cut-off to predict antigenic determinants (ADs), conformational epitopes (CEs) and sequential epitopes (SEs).	Conformational B Cell Epitope Prediction	[22]
14. NetMHC 2.2	Predicts binding of peptides to a number of different HLA alleles using artificial neural networks (ANNs) and weight matrices.	HLA Class I Epitope prediction.	[23]
15. MHCPred 2.0	**MHCPred **uses the **additive method **to predict the binding affinity of major histocompatibility complex (**MHC**) class I and II molecules and also to the Transporter associated with Processing (TAP). Allele specific Quantitative Structure Activity Relationship (**QSAR**) models were generated using partial least squares (PLS).	MHC Class I and II epitope prediction.	[24]
16. Bimas	Ranks potential 8-mer, 9-mer, or 10-mer peptides based on a predicted half-time of dissociation to HLA class I molecules. The analysis is based on coefficient tables deduced from the published literature by Dr. Kenneth Parker, Children's Hospital Boston.	HLA Class I Epitope prediction.	[25]
17. Propred	Predicts MHC Class-II binding regions in an antigen sequence, using quantitative matrices derived from published literature. It assists in locating promiscous binding regions that are useful in selecting vaccine candidates.	Promiscous MHC Class II epitope prediction.	[26]
18. AlgPred	Predicts allergens in query protein based on similarity to known epitopes, searching MEME/MAST allergen motifs using MAST and assign a protein allergen if it have any motif, search based on SVM modules and search with BLAST search against 2890 allergen-representative peptides obtained from Bjorklund et al 2005 and assign a protein allergen if it has a BLAST hit.	Allergen Prediction	[27]
19. Allermatch	Predicts the potential allergenicity of proteins by bioinformatics approaches as recommended by the Codex alimentarius and FAO/WHO Expert consultation on allergenicity of foods derived through modern biotechnology.	Allergen Prediction	[28]
20. WebAllergen	Predicts the potential allergenicity of proteins. The query protein is compared against a set of pre-built allergenic motifs that have been obtained from 664 known allergen proteins.	Allergen Prediction	[29]

### Database access and interface

**Figure 2:**
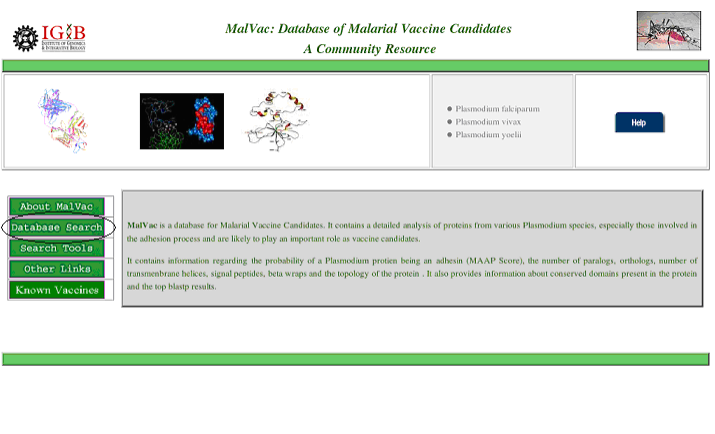
**The Home page of MalVac.** The "Database Search" facility can be used for first level search. Advanced search is provided in the "Search Tools" facility. "Other links" would take users to other websites of malaria for obtaining additional details and the "Known Vaccines" tab describes the details of the currently known vaccine candidates.

**Figure 3:**
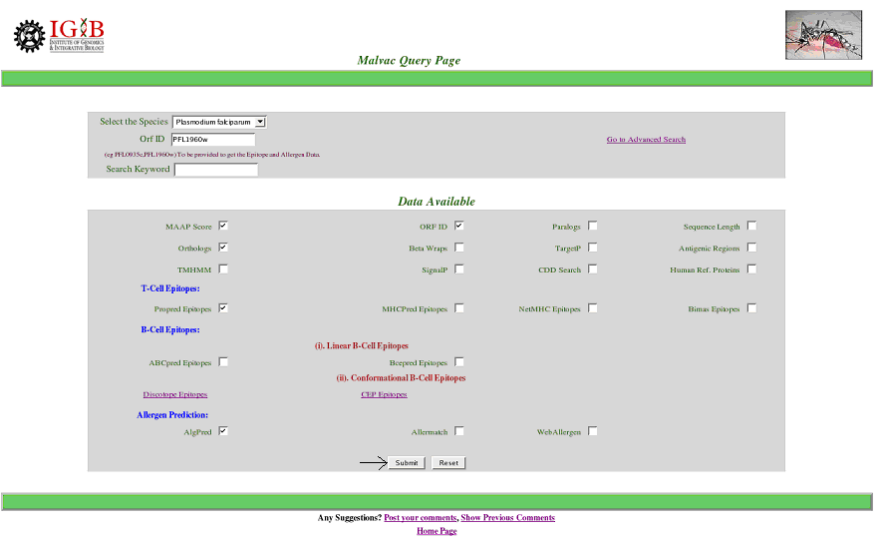
**The MalVac Query Page.** Default selections are MAAP score and ORF ID.

MalVac Database is freely available [32]. A user friendly web-based interface allows users to explore the site and fetch the data corresponding to their queries. For example, if the user needs to search database for data on a set of proteins given by their ORF identification tags one starts with clicking the "Database Search" button (Figure 2). This would take the user to the "MalVac Query Page". Here the user can search the database for adhesin proteins and their attributes corresponding to one or more ORF identification tags of a species or against a specific Keyword. To fetch the required data the corresponding checkboxes need to be toggled 'on' followed by clicking the submit button (Figure 3). The results are displayed in convenient tabular format and a facility to export the entire data has been provided. To get the Epitope and Allergen data the user must provide a specific ORF ID along with the species selected.

Advanced search facility of predicted malarial adhesins is also provided where the results can be filtered on the basis of Protein length, number of transmembrane spanning regions, localization and reliability class, presence or absence of betawraps, paralogs, orthologs, hits to Conserved Domain Database and Human Reference proteins (retrieved from NCBI through ftp on April 22, 2008). The results obtained can be exported by the user. The known vaccines link takes user to the page containing the list of known vaccine candidates provided in tabular form. This data can again be exported by the user. Facility to post comments by the user has been provided in MalVac web interface. Users can post their value added comments and suggestions on specific genes based on their own experience through the comment posting page of MalVac.

## Results and Discussion

MalVac Database contains analysis data on 332 potential vaccine candidates on three most important Plasmodium species. Of these, 161 are from *P. falciparum*, 137 are from *P. vivax *and 34 are from *P. yoelii*. First level of searching and retrieval of data is possible either through ORF ID or keywords. Multiple ORF IDs can be submitted using comma separation. Keywords can be used singly. If multiple keywords are used then the search is implemented using the AND Boolean. In the case of searching for epitope data, due to their huge size, data are conveniently retrieved in a singular mode for each ORF ID specifically. All data can be exported conveniently as a text file.

The database houses detailed information on these vaccine candidates analysed through 20 algorithms important from the view of reverse vaccinology. The analysis through these algorithms provide a broad range of information regarding Orthologs, Paralogs, BetaWraps, Localization, Transmembrane spanning regions, Signal Peptides, Conserved domains, similarity to Human Reference Proteins, T-cell epitopes, B-cell epitopes, Discotopes, and Allergen predictions.

Advanced level searches are also provided. In this facility users can search using combined feature selection. The most immediate application of such a scheme is in filtering for candidate proteins meeting a certain set of specifications. For example users formulate their queries by selecting for proteins that have less (or greater) than a specified number of transmembrane domains and less (or greater) than a specified length of protein. The features on which users can formulate their search could be based on Protein length, number of transmembrane spanning regions, localization-reliability class, presence or absence of betawraps, paralogs, orthologs, hits to CDD and human reference proteins in the advanced search page. The results obtained can be exported by the user.

## Conclusion

MalVac database was built as a community resource to aid malaria vaccinologists. MalVac is freely available with facility to export data and use for user's convenience [32].

## Competing interests

The authors declare that they have no competing interests.

## Authors' contributions

SR conceived the idea and provided guidance, suggestions, critical comments, and testing of MalVac. RG, SA, FAA, HVS collected data, organized systematically, prepared the codes for MalVac. SR and RG wrote the manuscript. All authors read and approved the final manuscript.
